# Low-level physical activity predictors among adults living with HIV in Ethiopia’s southern region, focusing on work, transportation, and recreation domains: *unmatched case-control study*

**DOI:** 10.1186/s13102-024-00860-2

**Published:** 2024-04-04

**Authors:** Girma Tenkolu Bune

**Affiliations:** School of Public Health (SPH), College of Medicine and Health Science (CMH), Dilla University (DU), Dilla, Ethiopia

**Keywords:** TPA, MLPA, HLPA, LLPA performance, PLWH, Cases, Controls, Dilla university, Gedeo Zone, Southern ethiopia, Ethiopia, And africa

## Abstract

**Background:**

Low-level physical activity (LLPA) is crucial for the well-being of adults living with HIV (PLWHs). However, many do not engage in enough physical activity, leading to adverse health outcomes. Identifying the determinants of LLPA can aid in developing effective interventions. Despite this, Ethiopia lacks evidence on this topic. This study aimed to identify predictors of LLPA among PLWHs in the Gedeo zone, located in southern Ethiopia.

**Methods:**

An unmatched case-control study was conducted on PLWHs in the Gedeo zone who visited two hospitals and healthcare institutions between December 29th, 2017 and January 22nd, 2019. Respondents were classified into three categories based on their total physical activity levels: high, moderate, and low. Cases were defined as those meeting the criteria for LLPA, while controls were those who did not fall under the cases category. Data was collected using the WHO Stepwise surveillance tool and analyzed using Epidata v3.1 templates and SPSS v22. Predictor variables with a P-value < 0.25 in bivariable analysis and < 0.05 with a 95% confidence interval in multivariable analysis were selected.

**Results:**

The study involved 633 HIV-positive adults, with a response rate of 92.41%. Most participants were under 34 years old, with an average age of 36.47±(9.055) for cases and 36.38±(8.389) for controls. The multivariable analysis revealed that educational status (AOR = 4.85, *P* = 0.02, 95%CI (1.28–18.44)), sex (AOR = 0.24, *P* = 0.04, 95%CI (0.07–0.90)), duration on ART being exposed for 1–4 Years (AOR = 0.12, *P* < 0.001, 95%CI (0.03–0.44)) and being exposed for 5–9 Years (AOR = 0.03, *P* < 0.001, 95%CI (0.01–0.16)), and former alcohol use (AOR = 0.11, *P* < 0.01, 95%CI (0.02–0.56) were significant predictors of LLPA performance.

**Conclusions:**

The study concluded that educational status, sex, ART duration, and past alcohol use are key determinants of LLPA performance among PLWHs in southern Ethiopia. This suggests that policymakers should implement public health campaigns to promote healthy habits, particularly low-level physical activity, among PLWHs.

**Supplementary Information:**

The online version contains supplementary material available at 10.1186/s13102-024-00860-2.

## Background

HIV/AIDS impacts 39.0 million people worldwide, leading to 40.4 million deaths and 630,000 fatalities. As of 2022, there are 25.6 million cases in Africa [1]. The World Health Organization (WHO), Global Fund, and UNAIDS are committed to eradicating the pandemic by 2030 and ensuring that 95% of patients have access to antiretroviral therapy (ART) by 2025 [2]. ART availability has enhanced life expectancy and quality of life for HIV-positive adults. However, non-communicable diseases (NCDs) like cardiovascular disease (CVD) pose obstacles to progress [3–5]. HIV-positive adults face a 1.5-2 times greater risk of CVD due to various factors, including comorbidities, infections, inflammation, medication side effects, metabolic issues, psychosocial concerns, and healthcare access disparities [4, 6]. This highlights the importance of scalable interventions, especially in HIV-endemic areas, [4] which should focus on lifestyle changes and physical activity [4, 6–8]. 

Physical activity (PA) is essential for adults living with HIV (PLWHs) as it helps prevent non-communicable diseases (NCDs), manage hypertension, maintain weight, and improve mental health [9]., However, many PLWHs do not engage in sufficient PA, which impacts their overall health [4–6]. The WHO recommends that healthy adults engage in 150 min of moderate-intensity activity, 75 min of vigorous-intensity activity, or a combination of both [9]. Accordingly, this study assessed the levels of PA among PLWHs in work, transportation, and recreation domains, categorized into high, moderate, and low PA levels. Low-level physical activity (LLPA) is defined as low-intensity, effort-intensive activity that promotes movement, burns calories, improves circulation, and reduces sedentary behavior, and not meeting the WHO criteria set to define high-level or moderate-level activity criteria [9–11]. LLPA is a minimally intense, effort-intensive activity that doesn’t significantly increase heart rate or cause sweating, excluding individuals who don’t meet moderate or high-level criteria.

LLPA is particularly beneficial for people living with HIV as it improves immune system function, cardiovascular health, muscular strength, mental well-being, reduces cardiovascular disease risk, and positively impacts metabolic profiles, muscle tissue, immune status, and overall quality of life [4, 6, 7]. However, recent evidence suggests that people living with HIV demonstrate lower LLPA performance contradicting the WHO Global Action Plan’s goal of a 15% decrease in inactivity rates by 2030 [4, 6, 7, 12, 13]., The WHO report reveals that 25% of the population fails to meet recommended physical activity levels, increasing the risk of death and contributing to 70% of productivity loss and disability expenses in low- and middle-income countries [14]. 

Most research on LLPA among people living with HIV has been conducted in high-income nations, with little study in HIV-endemic regions like Sub-Saharan Africa (SSA) [4–7]. For instance, Ethiopia lacks sufficient evidence of LLPA performance, with 39% of individuals having low physical activity levels and 53% not participating in regular exercise despite improved access to antiretroviral therapy [7]. Moreover, most SSA research uses non-standardized methodologies, leading to inconsistent results. Additionally, nearly all studies recommend physical activity programs as therapeutic strategies but fail to specify the type of physical activity, hindering effective solutions [4, 6, 7, 15]. 

This study aimed to identify predictors of LLPA among PLWHs in Gedeo zone, Ethiopia’s southern region. It fills a gap in literature and suggests policy direction to promote LLPA, raise awareness, provide exercise facilities, and integrate it into HIV care and treatment programs. The findings could inform evidence-based interventions to improve health outcomes in Ethiopia, where HIV and chronic diseases are prevalent. The study also contributes to the scientific community and serves as a foundation for future research.

## Methods

### Study contexts

This study was carried out in the Gedeo zone in the Southern Nations, Nationalities, and Peoples regions (SNNPRs). The area is situated 360 km south of Addis Ababa, the capital of Ethiopia, and 86 km south of Hawassa, the headquarters of the SNNPRs. The zone houses three main hospitals, one teaching and reference hospital, nine public health institutions comprising 141 health posts, and twenty-one private clinics. Some of the healthcare facilities, including five health centers and four hospitals, offer chronic HIV care services (CHCCs) funded by the CDC organization. During the study period, 3597 adult PLHIVs were registered in these public health institutions; 412 sought care from the ART clinics in public hospitals, and 2395 enrolled in clinics across all public health institutions. Antiretroviral treatment (ART) is accessible to PLWHs regardless of their CD4 level or clinical stage. Following a confirmed diagnosis, clinical assessment, and client readiness evaluation, rapid ART initiation should occur. Adults with advanced HIV or a CD4 count of ≤ 350 cells/mm3 must commence antiretroviral therapy (ART) promptly [10, 16]. 

### Study design and period

An unmatched case-control study was done between December 29th, 2017 and January 22nd, 2019.

### Source and study population

The study’s source population comprised confirmed PLWHs aged 18 and above who had previously been enrolled in chronic HIV care clinics in the Gedeo zone. These individuals had either returned to regular follow-up care or had registered for the first time and took part in a one-year survey to establish a database [10, 17]. The study population included PLWHs who met the conventional criteria for defining cases (LLPA performers) or controls (non-LLPA performers, such as MLPA and HLP performers), as well as the study’s inclusion and exclusion criteria for each comparison group [9]. 

### Outcome ascertainment

The study determined that moderate and vigorous intensity levels were at 4.0 and 8.0 for work, 4.0 for transportation, and 8.0 for recreational activities according to WHO criteria. The guidelines recommend at least 150 min of moderate-intensity physical activity, 75 min of vigorous-intensity exercise, or a combination of both each week, totaling 600 MET minutes to maintain health. The MET values and duration from TPA were classified as low, moderate, and high based on WHO’s comparative purpose criteria. Using these criteria, cases and controls among PLWHs were identified. Cases met the LLPA criteria, while controls included those classified as HLPA or MLPA [18]. 

### Case finding

#### Case identification

In this study, a case refers to a confirmed HIV + individual who took part in a previous survey and is now classified as an LLPA performer based on the study’s eligibility criteria, which were derived from prior databases [10, 17]. The study’s criteria for defining a case included the WHO’s previous recommendation for comparison [9]. This guideline defines a case or LLPA performer as a low-intensity, low-effort activity that does not greatly elevate heart rate or cause sweating and does not meet the standards for MLPA or HLPA categories. The study’s criteria for defining a case included the WHO’s former recommendation for comparison [9]. According to this guideline, a case or LLPA performer is a low-intensity, effort-intensive activity that does not significantly increase heart rate or sweat and does not meet MLPA or HLPA category standards.

### Case inclusion and exclusion criteria

This study involved PLWHS aged 18 or older, with or without antiretroviral treatment, who were registered in ART clinics of governmental healthcare institutions for an extended period or for the first time, and had previously participated in a survey and met the former case definition criterion of the WHO [10, 17]. It excluded PLWHs with missing or incomplete data from prior survey databases, which had a major influence on overall outcomes and risk variables.

#### Control identification

The study utilized the same criteria to select control subjects, which comprised HIV-positive individuals who had previously taken part in a survey and were included in a database. These individuals were now categorized as non-participants in LLPA (light or low-intensity physical activity) but regular participants in MLPA (moderate-intensity physical activity), HLPA (high-intensity physical activity), or both. According to WHO guidelines, HLPA involves vigorous-intensity activities on at least three days per week, totaling 1500 MET-minutes, or walking and engaging in moderate- to vigorous-intensity activities for seven days or more, achieving 3000 MET-minutes per week. Meanwhile, MLPA consists of vigorous-intensity activities on three or more days, lasting at least 20 min per day, as well as moderate-intensity activities or walking on five or more days, achieving a minimum of 600 MET-minutes per week [9]. 

### Control inclusion and exclusion criteria

The study used a cumulative case-control strategy [19], and applied the same eligibility criteria to select both controls and cases from the same sources. This included PLWHs who met the control definition criteria set by the WHO. However, the study excluded individuals of the same age group who had missing or incomplete data in previous survey databases.

### Sample size determination

The study’s sample size was determined using OpenEpi version 3 and several values, such as the confidence level, power, ratio of controls to cases, proportion of controls exposed, and important predictors of physical activity from previous Ethiopian research [15, 20]. After accounting for a 10% non-response rate, the total sample size was determined to be 576, utilizing the predictor factor of sex in Tegene et al. (2022) study [20]. However, to increase the power of the study, all participants who met the case and control eligibility requirements were included, resulting in a sample size of 633. This decision was based on the fact that the sample size calculated using the above assumptions was smaller than the sample size used in the previous survey. An additional file shows this in more detail [see Additional file 1. Sample size determination].

### Sampling procedure

The study enrolled participants from public healthcare organizations in the Gedeo zone based on factors such as patient size, quality of chronic HIV/AIDS medical care, and degree of service provision. Healthcare facilities were categorized based on the amount of care they provide, including hospitals and health centers. Through lottery methods, two hospitals (Yirga-Cheffe Primary Hospital and Dilla University Referral Hospital) and two healthcare centers (Dilla and Wonago Health centers) were randomly chosen. A survey was conducted to evaluate the movement of HIV patients to ART clinics at specific institutions using a proportionate allocation to sample size (PPS) approach to estimate the target sample size for each institution and then calculate the overall sample size for one year. Recruitment of PLWHs using systematic sampling procedures was undertaken until the required sample size for the survey was met [10, 17]. Lastly, the study used historical survey data and WHO guidelines to build an independent sample frame, and then used a cumulative case-control strategy and random sampling and lottery procedures to select and enroll cases and controls [19]. 

### Data collection methods and materials

The study, conducted in chronic HIV care clinics, involved four teams for data collection. Each team consisted of five data collectors and a supervisor, all of whom received training to ensure accurate data collection. Prior to the main study, a pretest was carried out on 5% of participants at an Ethiopian Kebado health center, which was not part of the study, to identify any issues with the questionnaire flow, language, interview duration, and other potential challenges [10, 17].

The WHO/NCD STEPS instrument 3.2 [21], validated in Ethiopian contexts [11], was utilized to assess NCD risk factors, along with a checklist to gather data from medical records. The questionnaire included demographic and behavioral data on respondents’ socioeconomic status, cigarette and alcohol consumption, and other healthy lifestyle-related information. A sixteen-item questionnaire was used to evaluate physical activity levels at work, during recreational activities, and when traveling. An additional file shows this in more detail [see Additional file 2. Instruments used] PLWHs were categorized into three groups based on their physical activity level: low, moderate, and high.(see Additional file 2 ).

The study also examined the prevalence of overweight, obesity, and elevated blood pressure using physical measurements such as height, weight, hip and waist circumferences, and blood pressure. Blood samples were collected from the antecubital fossa vein and stored at + 4 °C. The serum or plasma was then transported to Dilla University Hospital for biochemical analysis. Abnormal biochemical data was defined using the updated Adult Treatment Panel Three (ATP III) criteria cut-off reference [18]. 

### Data processing and analysis

The lead author collected the final hard copy data and entered it into Epidata v3.1 templates and then transferred it to SPSS v22 for analysis. Descriptive statistics, such as percentage, mean, and standard deviation, were computed to analyze biochemical and physical risk variables. The outcome variable was divided into two groups: cases and controls. The study employed bivariable binary logistic regression to find possible LLPA predictors. Variables with p-value < 0.25 were investigated further using multivariable logistic regression. The connection was statistically significant (P-value < 0.05) and analyzed using adjusted odds ratio, 95% confidence intervals, and P-value. Variance inflation factors (VIF) were used to assess multicollinearity, and the model’s fit was evaluated using a model summary, Omnibus test, Hosmer-Lemeshow goodness of fit test, and classification table. Quantitative exposure factors were categorized, and important explanatory variables were identified. The study employed various methods to handle missing data at each stage, ensuring no data was missing in the study.I’m sorry, I cannot fulfill that request.

### Variables and criteria used to operationalize

Socio-economic and demographic characteristics: The study analyzed socioeconomic and demographic factors, including age, education, and employment, to calculate a wealth index based on eleven household variables. The index was divided into four quintiles: lowest, medium, fourth, and fifth [16]. HIV/AIDS-related factors include antiretroviral therapy state, type of regimen, opportunistic infections, duration since diagnosis, duration on ART, functional status, current Tuberculosis state, and WHO staging (stage III and >/=III).Lifestyle and behavioral risk factors include smoking status, alcohol consumption status, daily Khat chew state, serving fruit/vegetables daily, oil type, and self-reported salt consumption. Smoking status, alcohol consumption status, and Khat chew state are also important factors to consider. Consuming too much, too much, just the right amount, or too little salt can impact overall health. Total Physical Activity (TPA) is the sum of all physical activities in healthy adult populations, including Work, Transportation, and Recreation, and can be measured using Metabolic Equivalents (MET) or percentages. MET: is the ratio of a person’s working metabolic rate relative to the resting metabolic rate. One MET is defined as the energy cost of sitting quietly, and is equivalent to a caloric consumption of 1 kcal/kg/hour. Moderate MET values were obtained for the Work, Transportation, and Recreational Activities Related domains. High-level physical activity (HLPA): involves at least three days of vigorous-intensity activity per week, achieving 1500 MET-minutes, or seven or more days of walking, moderate- or vigorous-intensity activities, achieving 3000 MET-minutes per week. Moderate-level physical activity (MLPA): is defined as three or more days of vigorous-intensity activity of at least 20 min per day, five or more days of moderate-intensity activity or walking for at least 30 min per day, or five or more days of a combination of walking, moderate- or vigorous-intensity activities achieving at least 600 MET-minutes per week. Low-level physical activity (LLPA): A person who does not meet HLPA or MLPA requirements. Not meeting WHO recommendations on physical activity for health: The percentage of respondents not meeting WHO recommendations on physical activity for health, which includes doing less than 150 min of moderate-intensity physical activity per week or equivalent. Total physical activity- mean: The mean minutes of total physical activity on average per day.; Domain specific physical activity- mean: Mean minutes spent in work-, transport- and recreation-related physical activity on average per day.; No physical activity by domain: Percentage of respondents classified as doing no work-, transport- or recreational related physical activity.; Composition of total physical activity: Percentage of work, transport and recreational activity contributing to total activity. The instrument questions for the above criteria cover work activities; travel to and from places, and recreational activities.No vigorous physical activity: The percentage of respondents who are not engaged in vigorous physical activity. The instrument inquiries included work activity and recreational activities; Sedentary practices; Minutes spent on average each day engaging in sedentary activities. The instrument question was Sedentary Behavior [9]. 

Anthropometric measurement related factors: elevated blood pressure, BMI, and waist circumference are related factors in anthropometric measurements, affecting BP, BMI, and WC measured based on the revised ATP criteria [16, 18]. Clinical history related factors: In order to analyze chronic illnesses in the last 12 months, inquire about the patient’s clinical history, including blood pressure, sugar, cholesterol, and cardiovascular history. Biochemical measurement; Elevated fasting plasma glucose (FBG>/=110 mg/dl), triglyceride (TGL>/=150 mg/dl), low high density lipoprotein (HDL_c < 40 mg/dl) and 50 mg/dl), total cholesterol (TC >/= 200 mg/dl), and low low density lipoprotein (LDL>/=150 mg/dl) were measured based on the revised ATP criteria. In general, using expectation and prior knowledge analysis approaches, the missing at random (MAR) assumptions were applied to manage missing interval/ratio and categorical data, respectively. However, no approaches were employed to investigate subgroup analysis [16, 18]. 

### Data quality control

The study followed established data quality control procedures at all stages, starting from the design phase. On-site training was provided to data collectors and supervisors to standardize processes. Quality control samples and instrument pre-testing were conducted at non-selected health facilities. Standard operating procedures were adhered to from sample collection to result reporting, with laboratory personnel handling all tasks. Additionally, direct supervisors and lead investigators monitored and followed up on each stage of the study to ensure its quality.

## Results

### Socio-economic and demographic characteristics

The study included 633 HIV-positive adults, with a 92.41% response rate. Most were under 34 years old, with an average age of 36.47+(9.06) for cases and 36.38+(8.39) for controls. It revealed that over half (53.4%) of LLPA performers were women, and the majority (63.0%) were from urban areas. In the study’s bi-variable analysis, a noteworthy association (*P* < 0.25) was found between low levels of physical activity and certain factors such as marital status, educational status, occupational status, wealth, and sex. (Table 1 attached at the end of the manuscript).

**Table 1:** Bivariable analysis, socio- demographic factors and LLPA, cases- controls, PLWHs, southern Ethiopia, 2023

Determinants	Low Level Physical Activity(LLPA)		COR (95% CI)	P-value
	Cases (*n* = 262)	Controls (*n* = 371)		
	No	No		
Age Groupsin year				
≤ 34	126	189	1	
35–44	88	115	1.15(0.80–1.64)	0.45
≥45	48	67	1.08(0.70–1.66)	0.75
Marital status				
Single	68	77	1	
Married	77	112	0.78(0.50–1.21)	0.26
Separated	48	87	0.62(0.39–1.01)	0.06***
Widowed	69	95	0.82(0.52–1.30)	0.40
Educational status				
No formal schooling	63	100	1	
Less than 1^0^ school	59	64	1.46(0.91–2.35)	0.12***
1^0^school completed	84	127	1.05(0.69–1.60)	0.82
2nd school completed	34	50	1.08(0.63–1.85)	0.78
College / University completed	22	30	1.16(0.62–2.20)	0.64
Occupation				
Government employed	41		1	
NGO-employed	12	43	0.84(0.35-2.00)	0.69
Self-employed	140	15	0.78(0.48–1.26)	0.31
Student	23	188	0.43(0.23–0.82)	0.01*
Homemaker	46	56	0.70(0.40–1.23)	0.01***
Wealth index in quintile				
Lowest	46	46	1	
second	36	62	0.58(0.32–1.04)	0.07***
Middle	85	117	0.73(0.44–1.19)	0.21***
Fourth	95	146	0.65(0.40–1.06)	0.08***
Sex				
Men	122	135	1	
Women	140	236	0.66(0.48–0.91)	0.01*
Place of residence				
Urban	165	241	1	
Rural	97	130	1.09(0.09–1.52)	0.20***

*Notes* *significant at *P* < 0.05 **Significant at *P* < 0.01 ***Significant at *P* < 0.25

Predictors: Age groups in year, Educational status, Ethnicity, Marital status, Occupation, Wealth index in quintile, sex, place of residence

Outcome: cases with low level physical activity (LLPA), control without LLPA

*Abbreviations* PLWHs, Adults living with HIV; 1^0^, primary; 2nd, secondary; COR, crude odd ratio

### HIV/AIDS-related factors

In this respect, study report was discovered that a greater proportion of participants involved in LLPA were undergoing antiretroviral therapy (ART), predominantly through first-line ART regimens. It also noted that viral RNA levels were elevated in cases when compared to control groups. Furthermore, the study revealed that factors including ART status, type of regimen, duration on ART, WHO staging, and other opportunistic infections (OI) besides TB significantly impacted low-level physical activity. (Table 2 attached at the end of the manuscript).

**Table 2:** Bivariable analysis, on HIV/AIDS-associated factors and LLPA, cases-controls, PLWHs, southern Ethiopia, 2023

Determinants	Low Level Physical Activity(LLPA)		COR (95% CI)	P-value
	Cases (*n* = 262)	Controls (*n* = 371)		
	No	No		
ART status				
ART-naive	84	127	1	
ART exposed	178	244	1.10(0.79–1.54)	0.06***
Type of ART regimen				
Non-in either of the two Therapy (Rx)^a^	84	127	1	
1st line	176	236	1.13(0.81–1.58)	0.49
2nd line	2	8	0.38(0.08–1.82)	0.23***
1st line ART regimen				
AZT-3TC-NVP	36	43	1	
AZT-3TC-EFV	18	17	1.27(0.57–2.81)	0.56
TDF-3TC-EFV	102	137	0.89(0.53–1.48)	0.65
TDF + 3TC + NVP	20	35	0.68(0.34–1.38)	0.29
ABC + 3TC + EFV	86	4	NC	
NA /Not applicable	36	135	0.76(0.45–1.28)	0.30
Duration since diagnosed with HIV				
< 1 year	33	55	1	
1–4 Years	208	275	1.26(0.79–2.01)	0.33
5–9 Years	21	41	0.85(0.43–1.69)	0.65
Duration since on ART				
< 1 year	55	16		
1–4 Years	48	113	0.12(0.06–0.24)	0.000**
5–9 Years	43	95	0.13(0.07–0.26)	0.000**
Functional status				
Ambulatory	73	99		
Working	189	272	0.94(0.66–1.34)	0.74
WHO staging				
< III stage	215	274		
≥ III stage	42	92	0.58(0.39–0.87)	0.009**
OI other than TB				
No	232	307		
Yes	30	64	0.62(0.39–0.99)	0.04*
Recent TB status				
No	223	318	1	
Yes	39	53	1.05(0.67–1.64)	0.83
CD4-levels cells mm3				
< 350	37	50	1	
350–500	64	84	1.03(0.60–1.76)	0.92
≥500	161	237	0.92(0.57–1.47)	0.72
Viral RNA levels > = 1500 copies/ml				
No	252	358	1	
Yes	10	13	0.92(0.40–2.12)	0.84

*Notes* *significant at *P* < 0.05 **Significant at *P* < 0.01 ***Significant at *P* < 0.25

Predictors: ART status, Type of ART regimen,1st line ART regimen, OI other than TB, Functional status, WHO staging, Viral RNA levels > = 1500 copies per ml, CD4-levels cells mm3, Duration since on ART, Duration since diagnosed with HIV

Outcome: cases with low level physical activity (LLPA), control without LLPA

*Abbreviations*: Rx–Therapy; HIV, human immunodeficiency virus; PLWH; people living with HIV, ART, antiretroviral therapy; 1st line regimen; 2nd line regimen; OI, opportunistic infections; TB, tuberculosis; WHO, world health organizations; RNA, Ribonucleic Acid; ml, milliliter; mm3, cubic millimeter; COR, crude odd ratio; AOR, Adjusted odd ratio

### Lifestyle/behavioral risk factors

The study revealed that 5.7% of active LLPA participants and 5.9% of control groups remembered the age when they began smoking, and the average age of smoking initiation was similar for both groups. The study also showed that a similar percentage of frequent Khat chewers were present in both groups. On average, PLWHs classified as LLPA performers consumed slightly more servings of fruits and vegetables than their control counterparts. However, a small percentage of individuals in both groups consumed fewer than five servings of fruits and vegetables each day. The results of the bivariable analysis indicated that various factors such as alcohol consumption, type of oil used at home, and self-reported salt consumption were linked to LLPA. (Table 3 at the end of the manuscript).

**Table 3:** Bivariable analysis, on behavioral risk factors and LLPA, cases- controls, PLWHs, southern Ethiopia, 2023

Determinants	Low Level Physical Activity(LLPA)		COR (95% CI)	P-value
	Cases (*n* = 262	Controls (*n* = 371)		
	No	No		
Smoking state				
Non-smoker	197	277	1	
All smoker	65	94	0.97(0.68–1.40)	0.88
All smoker				
Past smoker	25	39	1	
Current smoker	40	55	1.14(0.59–2.17)	0.70
Alcohol consumption status				
Lifetime abstainer	140	193		
Consumer	122	178	0.95(0.69–1.30)	0.73
Current alcoholic drinker(past 30 days)				
No	195	301	1	
Yes	67	70	1.48(1.01–2.16)	0.04*
Past alcoholic drinker (former drunker but not drank in the past 12 months)				
No	109	139	1	
Yes	13	39	0.43(0.22–0.84)	0.01*
Daily Khat chew state				
No	222	307	1	
Yes	40	64	0.86(0.56–1.33)	0.51
The average frequency of serving fruit/vegetables/ day				
No	253	351	1	
Yes	9	20	0.62(0.28–1.39)	0.24***
Type of oil				
Vegetable oil	18	24	1	
Butter	25	59	0.57(0.26–1.22)	0.15***
Margarine	80	82	1.30(0.66–2.58)	0.45
Solid fats	118	167	0.94(0.49–1.81)	0.86
Don’t know	21	39	0.72(0.32–1.61)	0.42
Self-reported consumption of salt				
Far too much	114	139	1	
Too much	105	151	0.85(0.60–1.21)	0.36
Just the right amount	29	62	0.57(0.34–0.95)	0.03*
Too little	14	19	0.90(0.43–1.87)	0.78

*Notes* *significant at *P* < 0.05 **Significant at *P* < 0.01 ***Significant at *P* < 0.1

Predictors: Smoking state, all smoker, Alcohol consumption status, All alcoholic consumer, Average frequency of serving fruit/vegetables/ day, Type of oil, Total physical activity state, Physical activity levels, High levels physical activity among physically active, Moderate physical activity, Low levels physical activity, Running sedentary life, Composited total physical activity from work, Composited total physical activity from transport, Composited total physical activity from recreation

Outcome: cases with low level physical activity (LLPA), control without LLPA

*Abbreviations* PLHIVs, people living with HIV; WHO, world health organizations; COR, crude odd ratio; AOR, Adjusted odd ratio; GROPAFA, global recommendation of physical activity for health

### Anthropometric, clinical, and biochemical related factors

The study focused on PLWH participants with elevated chronic diseases 12 months prior, specifically analyzing blood pressure, blood sugar, cholesterol, and cardiovascular disease in the context of LLPA. It used the criteria of the revised Adult Treatment Panel III to analyze PLWH anthropometric and biochemical levels. Based on this criterion, the study found that a significant percentage of PLWH participants in the LLPA group had increased blood pressure, BMI, and weight gain. The biochemical data also showed elevated levels of fasting plasma glucose, triglycerides, low HDL-c, high LDL-c, and total cholesterol. The binary logistic regression analysis identified a history of elevated chronic disease 12 months prior to the current study as a factor associated with LLPA, as well as high blood pressure measurement, high waist circumference, high triglyceride, and high total cholesterol. (Table 4 attached at the end of the manuscript)

**Table 4:** Bivariable analysis, anthropometric and biochemical factors and LLPA, cases-controls, PLWHs, southern Ethiopia, 2023

Determinants	Low Level Physical Activity(LLPA)		COR (95% CI)	P-value
	Cases (*n* = 262	Controls (*n* = 371)		
	No	No		
Patient previous 12 months chronic diseases elevated history				
Blood pressure				
No	241	322	1	
Yes	21	49	0.57(0.33–0.98)	0.04*
Blood sugar				
No	214	291	1	
Yes	48	80	0.52(0.28–0.96)	0.04*
Blood cholesterol				
No	247	358	1	
Yes	15	13	1.67 (0.78 − 0.58)	0.19***
Cardiovascular				
No	241	357	1	
Yes	21	14	2.22(1.11–4.46)	0.02*
Anthropometric measurements related variables #				
Blood pressure (BP-measure>/= 130/85 mmHg)				
No	240	331	1	
Yes	22	40	0.74(0.53–1.01)	0.06***
BMI (> 30 kg/M2)				
No	213	282	1	
Yes	49	89	0.76(0.44–1.31)	0.32
High WC >/= 102 CM for male and >/= 88 CM for female ATP criteria				
No	213	282	1	
Yes	49	89	0.73(0.49–1.08)	0.11***
Biochemical measurement related variables #				
Fasting plasma glucose >/=110				
No	192	274	1	
Yes	70	97	1.03(0.72 − 0.47)	0.87
Triglyceride (TGL levels >/=150 mg/dl				
No	157	241		
Yes	105	130	1.24(0.90–1.72)	0.20***
Low HDL_c < 40 mg/dl (male) and < 50 mg/dl(female)				
No	170	246		
Yes	92	125	1.07(0.76–1.49)	0.71
High TC >/= 200 mg/dl				
No	193	245		
Yes	69	126	0.70(0.49–0.99)	0.04*
High LDL>/=150 mg/dl				
No	149	209		
Yes	113	162	0.98(0.71–1.35)	0.89

*Notes* *significant at *P* < 0.05 **Significant at *P* < 0.01 ***Significant at *P* < 0.25

Predictors: Patient previous 12 months chronic diseases elevated history (Blood pressure, blood sugar, cholesterol, Cardiovascular disease), Anthropometric measurements related variables (BP, BMI, WC), and Biochemical measurement related variables (HDL, LDL, TGL, TC)

Outcome: cases with low level physical activity (LLPA), control without LLPA

*Abbreviations* AOR, Adjusted odd ratio; COR, Crude odd ratio, HLPA, High level of Physical activity, HDL, High density,lipoprotein PLWH, people living with HIV; WHO, world health organizations; COR, crude odd ratio; Total cholesterol, TGL, Triglycerides, LDL, Low density lipoprotein,

# All the anthropometric and biochemical measurements were compiled based on the revised Adult treatment panel three ( ATPIII ) criteria

### Total physical activity domains related result

The study revealed that a significant percentage of cases (40.8%) did not meet WHO health criteria compared to controls (22.4%). It also found that a higher percentage of LLPA performers (16.0%) met the requirements for physical activity for health compared to non-LLPA performers (3.2%). Transportation, work, and recreation were the main contributors to total physical activity in both cases and controls, with cases showing higher levels of physical activity in all three activity domains. According to the study, participants in the LLPA group engaged in physical activity for a shorter duration each day (46.31 ± 50.07 min) compared to their control counterparts (74.08 + 84.63 min). Additionally, both groups spent a significant amount of time on sedentary activities, with LLPA participants sitting for slightly less time on average (1513.13 ± 2490.71 min) compared to controls (1624.44 ± 2543.17 min).

### Predictors of low-level physical activity (LLPA)

The study indicates a significant connection between low level physical activity (LLPA) and various exposure variables with p-values under 0.25. These variables include marital status, education, occupational status, wealth index, sex, ART status, time on ART, WHO staging, OI other than TB, current or past alcoholic drinker, consuming less than five servings of fruit and vegetables per day, type of oil used at home, self-reported salt consumption, and previous history of elevated chronic diseases such as blood pressure, blood sugar, cholesterol, and cardiovascular diseases. However, due to multicollinearity issues, factors related to ART regimens were excluded from further analysis. The model’s suitability for further analysis was confirmed using a variety of tests, including the Summary − 2 Log-likelihood, Cox & Snell R Square, Nagelkerke R Square, Hosmer, and Lemeshow Test, as well as the overall percentage of Classification table findings. The results of the multivariable analysis indicated that educational status (AOR = 4.85, *P* = 0.02, 95%CI (1.28–18.44)), sex (AOR = 0.24, *P* = 0.04, 95%CI (0.07–0.90)), duration on ART (AOR = 0.12, *P* < 0.001, 95%CI (0.03–0.44)), exposure for 5–9 Years (AOR = 0.03, *P* < 0.001, 95%CI (0.01–0.16)), and past alcohol use (but not within the previous 12 months) (AOR = 0.11, *P* < 0.01, 95%CI(0.02–0.56)) were significant predictors of LLPA performance. (Table 5 attached at the end of the manuscript).

**Table 5:** Multivariable analysis, between all predictor and LLPA, cases- controls, PLWHs, southern Ethiopia, 2023

Determinants	Low Level Physical Activity(LLPA)	
	AOR(95%CI)	P-value
Marital status		
Single	1	
Married	1.55 (0.44–5.42)	0.50
Separated	0.68 (0.16–2.82)	0.60
Widowed	1.48 (0.39–5.70)	0.66
Educational status		
No formal schooling	1	
Less than primary school	1.65 (0.38–7.09)	0.50
Primary school completed	4.85 (1.28–18.44)	0.02*
Secondary school completed	2.52 (0.45–14.10)	0.29
College/University completed	1.21 (0.11–13.93)	0.88
Occupational status		
Government employed	1	
NGO-employed	0.07 (0.00-1.58)	0.09
Self-employed	0.58 (0.09–3.56)	0.56
student	1.29 (0.12–13.83)	0.83
Homemaker	1.19 (0.14–10.23)	0.87
Wealth index in quartiles		
Lowest	1	
second	0.94 (0.16–5.64)	0.94
middle	0.83 (0.16–4.45)	0.83
Fourth	0.30 (0.05–1.67)	0.17
Sex		
Men	1	
Women	0.24 (0.07–0.90)	0.04*
ART status		
ART naïve	1	
ART Exposed	3.23 (0.89–11.74)	0.07
Duration on ART		
< 1 year	1	
1–4 Years	0.12 (0.03–0.44)	0.001**
5–9 Years	0.03 (0.01–0.16)	0.001**
WHO staging		
< III		
>/=III	0.39(0.13–1.24)	0.11
OIs other than TB		
No		
Yes	0.81 (0.23–2.86)	0.75
Current (past 30 days) alcohol use state		
No		
Yes	1.22 (0.42–3.55)	0.72
Former Alcoholic drinker		
No		
Yes	0.11 (0.02–0.56)	0.01*
Less than five servings of fruit and/or vegetables on average per day		
No		
Yes	0.50 (0.01–20.73)	0.72
Type of Oil used at home		
Vegetable oil		
Butter	0.19 (0.02–1.58)	0.13
Margarine	0.48 (0.06–4.05)	0.50
Solid fats	0.69 (0.12–4.13)	0.69
Don’t know	0.41 (0.04–4.91)	0.48
Self-reported frequency of salt or salty sauce consumed		
Far too much		
Too much	0.48 (0.17–1.33)	0.16
Just the right amount	0.40 (0.09–1.86)	0.24
Too little	2.58 (0.30-21.92)	0.38
History of diagnosed with elevated BP in the past 12 months		
No		
Yes	0.27 (0.07–1.12)	0.07
History of diagnosed with elevated Blood sugar in the past 12 months		
No		
Yes	1.14 (0.37–3.52)	0.83
History of diagnosed with elevated Blood cholesterol in the past 12 months		
No		
Yes	3.31 (0.42–26.19)	0.26
History of diagnosed with elevated CVDs in the past 12 months		
No		
Yes	3.92 (0.67–22.90)	0.13
High blood pressure (BP-measure>/= 130/85 mmHg)		
No		
Yes	0.81 (0.29–2.25)	0.69
High waist circumference (WC >/= 102 CM(male) and >/= 88 CM (female)		
No		
Yes	0.65 (0.15–2.88)	0.57
Triglyceride (TGL levels >/=150 mg/dl)		
No		
Yes	1.56 (0.54–4.50)	0.41
Low level of HDL_c (Measured HDL_c < 40 mg/dl (male) and < 50 mg/dl(female))		
No		
Yes	1.45 (0.51–4.12)	0.49

*Notes* *significant at *P* < 0.05 **Significant at *P* < 0.01

Predictors: Marital Status, Educational status, Occupational status, Wealth in quartiles, Sex, Duration since diagnosed with HIV, Total duration on ART, WHO Staging, OI other than TB, Former drinkers stopped drinking due to health reasons state (not used in the past 12 months), Less than five servings of fruit and/or vegetables on average per day, Type of oil or fat used for cooking at home, History of diagnosed with elevated Blood pressure in the past 12 months, History of diagnosed with elevated Blood sugar in the past 12 months, History of diagnosed with elevated blood cholesterol level in the past 12 months, Previous history of diagnosed with CVDs, Blood pressure measurement levels (BP >/= 130/85 mmHg), High level of waist circumference (WC >/= 102 CM for male & >/= 88 CM for female ATP criteria), Plasma triglyceride levels (TGL>/=150 mg/dl), Type of ART regimen, None of the two regimen type

Outcome: cases with low level physical activity (LLPA), control without LLPA

*Abbreviations* ATP; Adult treatment panel, BP, Blood Pressure, CM, Centimeter, CVDs, Cardiovascular disease, mmHg, millimeter of mercury, HLPA, High level of Physical activity, HDL, High density,lipoprotein PLWH, people living with HIV; ART, antiretroviral therapy; OI, opportunistic infects; TB, tuberculosis; TC, Total cholesterol, TGL, Triglycerides, LDL, Low density lipoprotein, WHO, world health organizations; AOR, Adjusted odd ratio, 1^0^, primary; 2nd, secondary, WC, Waist Circumference,

**#** All the anthropometric and biochemical measurements were compiled based on the revised Adult treatment panel three ( ATPIII ) criteria

## Discussion

The aim of this study was to determine the factors that can predict low-level physical activity (LLPA) performance in adult HIV patients in the Gedo zone of Southern Ethiopia. The study found that educational status, sex, duration of antiretroviral treatment (ART), and past alcohol use were all significant predictors of LLPA performance.

In a study, it was found that people with HIV who finished primary school and received education were almost five times more likely to participate in regular LLPA compared to those who did not. Further research by Tekalegn et al.(2022) in Ethiopia [15] and Mabweazara SZ, et al. (2021) in South Africa [22]. also indicated a strong connection between education and consistent physical activity. This could be attributed to the fact that education improves health literacy, thereby enhancing participation in physical activity and access, with higher levels of education providing more resources and opportunities for this [23]. However, some earlier research did not find a statistically significant correlation between education and regular physical activity attendance [20]. Additionally, a recent study in Cameroon [24] similarly found no link between the educational status of individuals living with HIV and regular physical activity performance, suggesting that these factors are independent. The discrepancies may stem from variations in measurement and designs considered in the former and latter papers. While the current study utilized comprehensive physical activity measurement tools and a case-control study approach to assess regular physical activity among individuals living with HIV, the latter used a cross-sectional study design to measure overall physical activity using different tools. Furthermore, differences in socioeconomic factors, cultural changes, and shifts in the educational system between the studies conducted before and the present ones may explain the observed variations. However, to gain a deeper understanding, future research should focus on health literacy, self-efficacy, resource availability, and social support networks.

This study found that women were 76% less likely to engage in LLPA than men, consistent with other research showing lower participation in physical exercise among women [20, 25–27]. This might be because HIV/AIDS women often face challenges like cultural differences, gender traditions, caregiving responsibilities, safety concerns, and restricted recreational opportunities, leading to low self-esteem and suicidal thoughts [25]. However, some studies in Ethiopia [15], Uganda [4], Malawi [28], and Cameroon [24] have found the opposite, with women more likely to participate in LLPA than men. These differences may be attributed to varying study designs, sample characteristics, cultural backgrounds, or measurement methodologies. The larger sample size in the present study could also account for these variations. Nonetheless, the findings underscore the need for further research into gender differences in LLPA participation and suggest policy implications for gender equity, such as gender-sensitive approaches, addressing social barriers, providing safe spaces, tailored programming, and educational campaigns in sports and leisure activities.

The study also revealed a notable decline in low-level physical activity (LLPA) among people living with HIV (PLWHs) who had been undergoing antiretroviral therapy (ART) for 1–4 years and 5–9 years. Another investigation by Tegene, Yadessa, et al. (2022) discovered that individual on ART for 24 months or more were nearly twice as likely to be physically inactive compared to those on ART for less than 24 months [20]. Similarly, a study by Chisati et al. (2020) from Malawi found that PLWHs on antiretroviral therapy for 1–3 years and over 3 years experienced a significant decrease in LLPA performance [28]. These parallels may be attributed to the physical side effects of long-term ART, such as fatigue and muscle weakness, which can hinder PLWHs from engaging in LLPA and may lead to HIV-related comorbidities, restricting participation and diminishing motivation for LLPA among PLWHs due to the psychological impact of long-term treatment. The study proposes that healthcare providers should establish peer support programs, conduct regular follow-ups, and raise awareness to increase LLPA participation and improve overall health. It also carries policy implications such as enhancing LLPA, reducing barriers, and investigating factors associated with ART duration, depression, social support, and self-efficacy. Furthermore, the study underscores the importance of further research in the area to comprehend the specific factors contributing to the observed decrease in LLPA among PLWHs with prolonged ART exposure in Ethiopia’s southern region.

Moreover, the study found that people living with HIV who had a history of alcohol consumption were 89% less likely to participate in low-level physical activity. This is consistent with earlier research showing a detrimental link between past alcohol use and low-level physical activity [29]. This could be attributed to alcohol exacerbating sedentary behavior, decreasing physical activity, and contributing to unhealthy lifestyle choices. It may also be linked to earlier studies associating alcohol consumption with poorer LLPA performance and an elevated risk of injury. However, our research contradicts prior studies that suggested a link between consistent low-level physical activity performance and health benefits [20, 23, 30] and a scoping review revealed no association between alcohol consumption and PA involvement [27]. The discrepancies noted between current and prior studies may arise from variations in community attitudes, participant characteristics, methodological approaches, measurement techniques, and outcome determination methods. For example, our study was conducted among PLWHs in Gedeo zone, a semi-urban area in the southern regions with restricted social values towards alcohol use. We used an unmatched case-control design, comprehensive assessment tools, and standardized WHO criteria to determine the LLPA. In contrast, the previous studies employed a cross-sectional, qualitative, and scoping review method to assess total physical activity levels among PLWHs with diverse demographic traits, including place of residence and cultural disparities, which may be considered as a justification. Despite the observed differences, overall the study finding recommends that healthcare providers consider alcohol consumption when advising HIV patients on engaging in low-level physical activity, and that public health initiatives focus on reducing alcohol intake and promoting physical activity. Further research should explore strategies to encourage low-level physical activity participation in HIV patients with a history of alcohol use.

Finally, the current study may have limitations such as selection bias, recall bias, measurement bias, and confounding factors, which may stem from the nature of an institution-based study, its design, and method of data collection. To address these limitations, the study involved healthcare institutions that accounted for over 90% of the enrolled PLWHs in the region, selected participants carefully, used standardized measurement tools supplemented with show cards, and employed appropriate statistical methods to control for confounding variables. These measures could enhance the study’s robustness and the reliability of its results, and may also prove valuable for future research. Nonetheless, it is important to acknowledge these limitations when utilizing the study’s findings.

## Conclusion

The study found that educational status, gender, ART duration, and past alcohol use are important factors in low-level physical activity (LLPA) among people living with HIV (PLWHs) in Ethiopia’s southern Gedeo zone. This indicates that policymakers should prioritize public health campaigns promoting healthy practices, particularly regular LLPA, and integrating self-management into HIV treatment programs. Educational programs should also improve health literacy and LLPA awareness. Additionally, healthcare practitioners should offer personalized counseling to increase LLPA participation, especially for those on long-term antiretroviral therapies or with a history of alcohol use. Community-based programs should promote LLPA performance and educate about the benefits of healthy living. Further, matched case-control and longitudinal studies are needed to establish the link between LLPA and HIV-related outcomes, and to explore the connection between LLPA performance, gender disparities, ART duration, and previous alcohol consumption. 

### Electronic supplementary material

Below is the link to the electronic supplementary material.

Supplementary Material 1

## Data Availability

All data generated or analyzed during this study are included in this manuscript and uploaded tables.

## Declarations

### Guideline

The Strengthening the Reporting of Observational Studies in Epidemiology (STROBE) statement: guidelines for reporting observational studies.(HYPERLINK "#_ENREF_31"31)

## Abbreviations

AOR: Adjusted Odd Ratio
AIDS: Acquired Immunodeficiency diseases
LLPA: Low level physical activity
MET: Metabolic equivalent time
MLPA: Moderate level physical activity
HIV: Human Immune Virus
COR: Crude Odd Ratio
PA: Physical activity
PLWHs: Adults living with HIV Human Immune Virus
HLPA: High level physical activity
TPA: Total physical activity
WHO: World Health Organization, UNIDs
