# Supplementary material for: Low-level physical activity predictors among adults living with HIV in Ethiopia’s southern region, focusing on work, transportation, and recreation domains: unmatched case-control study
Source: BMC Sports Sci Med Rehabil. 2024 Apr 4;16:78. doi: 10.1186/s13102-024-00860-2 (PMC10993537; doi:10.1186/s13102-024-00860-2)
Supplement: Supplementary file 1 — Supplementary Material 1 [file 13102_2024_860_MOESM1_ESM.pdf]

# The STROBE guidelines


## ABSTRACT

An observational study is a type of epidemiological study design, which can take the form of a cohort, a case–control, or a cross-sectional study. When presenting observational studies in manuscripts, an author needs to ascertain a clear presentation of the work and provide the reader with appropriate information to enable critical appraisal of the research. The Strengthening the Reporting of Observational studies in Epidemiology (STROBE) guidelines were created to aid the author in ensuring high-quality presentation of the conducted observational study. The original articles publishing the STROBE guidelines together with their bibliographies were identified and thoroughly reviewed. These guidelines consist of 22 checklist items that the author needs to fulfil before submitting the manuscript to a journal. The STROBE guidelines were created to aid the authors in presenting their work and not to act as a validation tool for the conducted study or as a framework to conduct an observational study on. The authors complying with these guidelines are more likely to succeed in publishing their observational study work in a journal.


**Key words:** Data reporting; epidemiology; observational studies; publishing; research design

## Introduction

Different epidemiological study designs are available and are adopted by a researcher depending on the research question at hand. An observational study is one type of epidemiological study design, which can be in the form of a cohort, a case–control, or a cross-sectional study. This type of study design (observational) is defined as a nonexperimental research, where the researcher observes a particular environmental behavior without artificially controlling the environment under study. To ascertain high-quality reporting of observational studies, the Strengthening the Reporting of Observational studies in Epidemiology (STROBE) guidelines were developed following a collaborative initiative of epidemiologists, methodologists, statisticians, researchers, and journal editors in 2004.[1] These guidelines were created to aid in the presentation of the conducted observational study to ensure adequate reporting (what was planned, done, found, and concluded) as well as assessment of the strengths and weaknesses of the study.[1,2] Such study information is of vital importance in a manuscript since this will determine whether the established results can be included in systemic reviews later on.[3,4] Furthermore, the STROBE guidelines enable the journal's editor, reviewers, and the readers to critically appraise the study.[1]

## The STROBE Guidelines

The aim of the STROBE guidelines was to provide a readily available checklist to ensure a clear presentation of what was planned and conducted in an observational study. These studies are set out to investigate the associations


| Access this article online | |
|---|---|
| **Website:** www.saudija.org | Quick Response Code |
| **DOI:** 10.4103/sja.SJA_543_18 | |


This is an open access journal, and articles are distributed under the terms of the Creative Commons Attribution-NonCommercial-ShareAlike 4.0 License, which allows others to remix, tweak, and build upon the work non-commercially, as long as appropriate credit is given and the new creations are licensed under the identical terms.

**For reprints contact:** reprints@medknow.com

**How to cite this article:** Cuschieri S. The STROBE guidelines. Saudi J Anaesth 2019;13:S31-4.

**Sarah Cuschieri**

Department of Anatomy, Faculty of Medicine and Surgery, Biomedical Building, University of Malta, Msida, Malta

**Address for correspondence:** Dr. Sarah Cuschieri, Department of Anatomy, Faculty of Medicine and Surgery, Biomedical Building, University of Malta, Msida MSD 2080, Malta. E-mail: sarah.cuschieri@um.edu.mt

between an exposure and a health outcome. In no way were these guidelines established to provide a methodological framework for conducting an observational study.[1] Nor were the guidelines developed as an instrument for quality evaluation of observational research.[2] Furthermore, the guidelines were not aimed to bring forward standardization of manuscripts but rather to encourage the production of interesting and narrative articles while maintaining transparency.[5]

## The Strobe Checklist

A total of 22 checklist items contribute to the STROBE guidelines. Eighteen items are common to all the three observational designs, that is, cohort, cross-sectional, and case–control studies. However, the remaining four checklist items (items number 6, 12, 14, and 15) have specific variations according to the study design. Table 1 exhibits the STROBE guidelines as published by Vandenbroucke *et al*.[1] The following is an abbreviated explanation of the checklist items.

## Item 1: Title and Abstract

The adopted study design should be part of the manuscript title to ensure correct indexing of the manuscript in electronic databases. Indexing of the published manuscript is of utmost importance to ensure visibility of a researcher's work and increase the citation potential of the published manuscript. Citation of published manuscripts is imperative for the enhancement of the researcher's research metrics and for increasing the prestigious acknowledgement of the researcher and his or her work within the scientific community.[6] The abstract should include a brief summary of the study and present only information found within the actual body of the manuscript.

## Items 2 and 3: Introduction

The introduction should consist of background information that will set the scene for the study and the objective of the study. The objective states the researcher's intentions for conducting the study and potential hypotheses that may arise from such work.

## Items 4–12: Methods

The methods section should provide a clear description of the study design at an early stage. This will enable the reader to understand the basis of the study and be able to critically appraise the study's methodology. The STROBE guidelines do not allow the use of the words "prospective" or "retrospective" or "concurrent" or "historical," but rather encourage the researcher to describe the actual methodology.[1]

Information on the tools of measurement, setting, and locations should be reported to enhance the reader's understanding of the study's results. The reporting of the participants' recruitment procedure will vary depending on the type of observation design being conducted. Therefore, it is important that the researcher is knowledgeable about the epidemiological methodological design for each different observational study (i.e., cohort, case–control, or cross-sectional, respectively).

All the variables considered for the descriptive and statistical analysis of the study need to be noted down within the methods section. This also includes the reporting of any specific cut-off points implemented during the analysis. It is essential that any exposures, confounders, or outcomes measurements are accounted for and reported for the reader to critically appraise the study's reliability and validity. The inclusion and exclusion criteria and methods to overcome any potential bias should be noted down as well.

The method used to establish the study size needs to be reported along with the confidence intervals considered. This is essential for the reader to ascertain whether sufficient statistical precision has been attained in the study.[1]

The reporting of statistical analysis will vary depending on the study design (i.e., cohort, case–control, or cross-sectional). However, it is important that all statistical methods and adjustments for potential confounders or missing data are noted down clearly.

## Items 13–17: Results

The results section should give an in-depth account of the response rate and the description of the study population along with the main descriptive and analytical results. The information provided will depend on the type of observational design (i.e., cohort, case–control, or cross-sectional) followed by the researcher and the corresponding statistical analysis performed.

## Items 18–21: Discussion

The discussion should address all the central issues of the study including the validity of the study. The objective/s of the

**Table 1: STROBE guidelines**

| STROBE guidelines | | |
|---|---|---|
| **Section/topic** | **Item number** | **Recommendation** |
| Title and abstract | 1 | Indicate the study's design with a commonly used term in the title or the abstract |
| | | Provide in the abstract an informative and balanced summary of what was done and what was found |
| Introduction | | |
| Background/rationale | 2 | Explain the scientific background and rationale for the investigation being reported |
| Objectives | 3 | State specific objectives, including any prespecified hypotheses |
| Methods | | |
| Study design | 4 | Present key elements of study design early in the manuscript |
| Setting | 5 | Describe the setting, locations, and relevant dates, including periods of recruitment, exposure, follow-up, and data collection |
| Participants | 6 | Cohort study – give the eligibility criteria, and the sources and methods of selection of participants; describe methods of follow-up<br>Case–control study – give the eligibility criteria, and the sources and methods of case ascertainment and control selection; give the rationale for the choice of cases and controls<br>Cross-sectional study – give the eligibility criteria, and the sources and methods of selection of participants |
| | | Cohort study – for matched studies, give matching criteria and number of exposed and unexposed<br>Case–control study – for matched studies, give matching criteria and the number of controls per case |
| Variables | 7 | Clearly define all outcomes, exposures, predictors, potential confounders, and effect modifiers; give diagnostic criteria, if applicable |
| Data sources/measurement | 8* | For each variable of interest, give sources of data and details of methods of assessment (measurement); describe comparability of assessment methods if there is more than one group |
| Bias | 9 | Describe any efforts to address potential sources of bias |
| Study size | 10 | Explain how the study size was arrived at |
| Quantitative variables | 11 | Explain how quantitative variables were handled in the analyses; if applicable, describe which groupings were chosen and why |
| Statistical methods | 12 | Describe all statistical methods, including those used to control for confounding |
| | | Describe any methods used to examine subgroups and interactions |
| | | Explain how missing data were addressed |
| | | Cohort study – if applicable, explain how loss to follow-up was addressed<br>Case–control study – if applicable, explain how matching of cases and controls was addressed<br>Cross-sectional study – if applicable, describe analytical methods taking account of sampling strategy |
| | | Describe any sensitivity analyses |
| Results | | |
| Participants | 13* | Report numbers of individuals at each stage of study – e.g., numbers potentially eligible, examined for eligibility, confirmed eligible, included in the study, completing follow-up, and analyzed |
| | | Give reasons for nonparticipation at each stage |
| | | Consider use of a flow diagram |
| Descriptive data | 14* | Give characteristics of study participants (e.g., demographic, clinical, social) and information on exposures and potential confounders |
| | | Indicate number of participants with missing data for each variable of interest |
| | | Cohort study – summarize follow-up time (e.g., average and total amount) |
| Outcome data | 15* | Cohort study – report numbers of outcome events or summary measures over time |
| | | Case–control study – report numbers in each exposure category, or summary measures of exposure |
| | | Cross-sectional study – report numbers of outcome events or summary measures |
| Main results | 16 | Give unadjusted estimates and, if applicable, confounder-adjusted estimates and their precision (e.g., 95% confidence interval); make clear which confounders were adjusted for and why they were included |
| | | Report category boundaries when continuous variables were categorized |
| | | If relevant, consider translating estimates of relative risk into absolute risk for a meaningful time period |
| Other analyses | 17 | Report other analyses done – e.g., analyses of subgroups and interactions, and sensitivity analyses |
| Discussion | | |
| Key results | 18 | Summarize key results with reference to study objectives |
| Limitations | 19 | Discuss limitations of the study, taking into account sources of potential bias or imprecision; discuss both direction and magnitude of any potential bias |
| Interpretation | 20 | Give a cautious overall interpretation of results considering objectives, limitations, multiplicity of analyses, results from similar studies, and other relevant evidence |
| Generalizability | 21 | Discuss the generalizability (external validity) of the study results |

*Contd...*

**Table 1: Contd...**

| STROBE guidelines | | |
|---|---|---|
| **Section/topic** | **Item number** | **Recommendation** |
| Other information | | |
| Funding | 22 | Give the source of funding and the role of the funders for the present study and, if applicable, for the original study on which the present article is based |

*Give information separately for cases and controls in case–control studies and, if applicable, for exposed and unexposed groups in cohort and cross-sectional studies

study should be kept in mind while discussing the findings. Comparisons to already published literature are essential. It may be appropriate for the discussion section to be subdivided into different sections to enable better interpretation of the study findings. The researcher should provide an objective assessment of the findings and avoid overinterpretations. Potential confounder effects that might have had an effect on the results and associations obtained in the study should be considered. Therefore, it is imperative to note down potential limitations faced by the study, while noting any bias that might have been present. Furthermore, researchers have to keep in mind that causality of a particular outcome cannot be established in most study designs, unless a longitudinal cohort study has been conducted. Therefore, this fact needs to be acknowledged during the discussion and may act as a study limitation for certain study designs. Study limitations go hand in hand with recommendations for further research to validate the study or further establish associations that were revealed by the study.

## Item 22: Funding and Sponsorship

The source of funding and the role of the funders in the study are essential pieces of information that are required at the end of the article. This is accompanied with any conflict of interest of both the author/s and the funders.

## Conclusion

A substantial number of journals are requesting authors to follow the STROBE guidelines before submitting their observational-study-inspired manuscript. Having a thorough understanding of the STROBE guidelines is therefore becoming a requisite for authors who wish to conduct and publish an observational study. These guidelines have been formulated as an aid to authors to enable them to construct an adequately presented manuscript that allows the reader to fully comprehend and critically appraise the manuscript.

### Financial support and sponsorship

Nil.

### Conflicts of interest

There are no conflicts of interest.

## References

1. Vandenbroucke JP, von Elm E, Altman DG, Gøtzsche PC, Mulrow CD, Pocock SJ, *et al.* Strengthening the reporting of observational studies in epidemiology (STROBE): Explanation and elaboration. PLoS Med 2007;4:e297.
2. von Elm E, Altman DG, Egger M, Pocock SJ, Gøtzsche PC, Vandenbroucke JP, *et al.* Strengthening the reporting of observational studies in epidemiology (STROBE) statement: Guidelines for reporting observational studies. BMJ 2007;335:806-8.
3. Jüni P, Altman DG, Egger M. Systematic reviews in health care: Assessing the quality of controlled clinical trials. BMJ 2001;323:42-6.
4. Egger M, Schneider M, Davey Smith G. Spurious precision? Meta-analysis of observational studies. BMJ 1998;316:140-4.
5. Schriger DL. Suggestions for improving the reporting of clinical research: The role of narrative. Ann Emerg Med 2005;45:437-43.
6. Cuschieri S. WASP (Write a scientific paper): Understanding research metrics. Early Hum Dev 2018;118:67-71.
